# miRviewer: a multispecies microRNA homologous viewer

**DOI:** 10.1186/1756-0500-5-92

**Published:** 2012-02-13

**Authors:** Adam Kiezun, Shay Artzi, Shira Modai, Naama Volk, Ofer Isakov, Noam Shomron

**Affiliations:** 1 Division of Genetics, Department of Medicine, Brigham and Woman's Hospital, Harvard Medical School, Boston MA 02115, USA; 2 Program Analysis and Transformation Group, IBM, Watson Research Center, Hawthorne, NY 10532, USA; 3 Department of Cell and Developmental Biology, Sackler Faculty of Medicine, Tel Aviv University, Tel Aviv 69978, Israel

## Abstract

**Background:**

MicroRNAs (miRNAs) are short non-coding RNAs that regulate gene expression via binding to the 3' ends of mRNAs. MiRNAs have been associated with many cellular events ascertaining their central role in gene regulation. In order to better understand miRNAs of interest it is of utmost importance to learn about the genomic conservation of these genes.

**Findings:**

The miRviewer web-server, presented here, encompasses all known miRNAs of currently fully annotated animal genomes in a visual 'birds-eye' view representation. miRviewer provides a graphical outlook of the current miRNA world together with sequence alignments and secondary structures of each miRNA. As a test case we experimentally examined the expression of several miRNAs in various animals.

**Conclusions:**

miRviewer completes the homologous miRNA space with hundreds of unreported miRNAs and is available at: http://people.csail.mit.edu/akiezun/miRviewer

## Findings

The gene expression pathway is very often regulated at the post-transcriptional level by small non-coding RNAs termed microRNAs (miRNAs) [1]. MiRNAs are 22 nucleotides long molecules that bind the 3' untranslated (3' UTR) region of the mRNA leading to facilitated mRNA degradation or inhibition of translation [2]. MiRNAs are pivotal regulators in many cellular processes such as development and differentiation (for example see [3]). Each miRNA has on average 200 conserved targets identified by miRNA:target sequence complementarity [4]. The miRNA genes are transcribed mostly by RNA polymerase II and are either expressed from intergenic regions or as part of a coding transcript when located in introns [4]. Most miRNAs were generated via genomic duplication events, though other events, such as through repetitive genomic sequences, were also observed. The unique evolutionary features of miRNAs are their high gain versus loss ratio and their resistance to changes or mutations particularly in the mature and functional targeting "seed" region [5].

In order to better understand a miRNA of interest, whether to learn about its genomic evolution, its potential target genes in various organisms, or in order to select a model animal to work on, it is important to comprehend its evolutionary conservation. Here we present the miRviewer web-server, which provides a comprehensive view of all known miRNAs in annotated animal genomes. The easily assessable data represents the miRNA gene repertoire in 50 animals. The web interface provides the user a simple and graphic view of the miRNA world. Additional layers of information, such as conservation, multiple alignments of the sequences, indication of mutated miRNAs, genomic loci and secondary structure, are also available.

## Implementation

**Figure 1:**
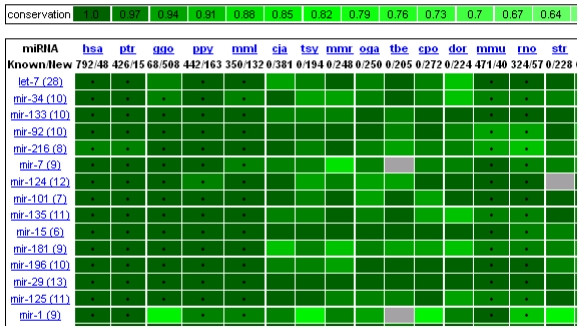
**A partial screenshot of miRviewer's main entry page**. Species are indicated on the X-axis while miRNAs are on the Y-axis. Level of conservation is indicated in shades of green and numerical values are indicated on top. Current sorting is human centric and is ordered by most to least conserved miRNAs. A dot at the center of each box indicates the presence of the miRNA in miRbase [6]. Lack of such dot indicates the identification of the miRNA gene through miRNAminer [7].

**Figure 2:**
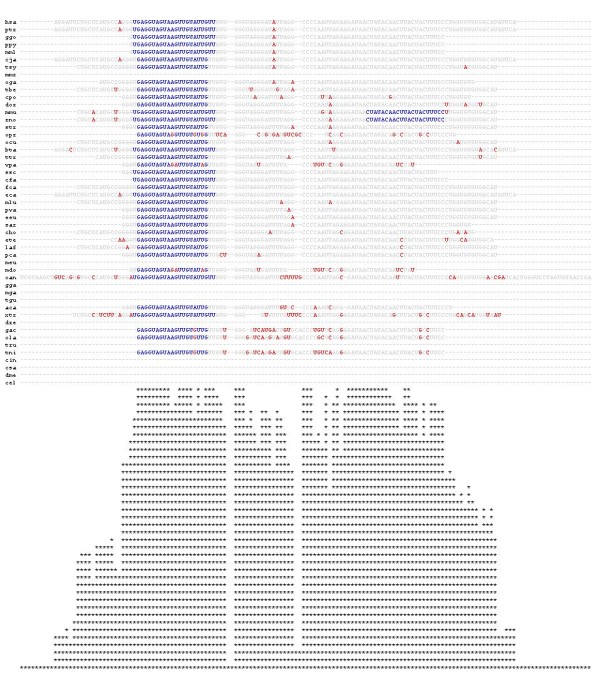
**A screenshot of miR-98a multiple alignment in different organisms**. Though this magnification does not allow reading the exact sequences, it does allow viewing each row representing miR-98a from a different animal. Mature (blue), mutation (red) and all other sequences (gray) are indicated (also see text). On the right are links to miRbase, genomic locations on UCSC Genome Browser [9] and Ensembl [8]. Below the alignments, conservation of the pre-miRNA sequence is plotted.

miRviewer presents homologous miRNAs that are either included in miRbase v.16 [6] or were identified by a full cross-search using miRNAminer [7]. Specifically, for each miRNA in miRbase v.16 a search was performed in all animal genomes in Ensembl 57 [8], using default miRNAminer settings. The most significant match identified was used in creating the current miRviewer web-server (Figures 1 and 2).

The candidate miRNAs identified by our miRNAminer method [7] were recognized using the following parameters: (i) Use BLAST [http://www.ncbi.nlm.nih.gov/BLAST] to find matches in target genomes (the whole precursor miRNA from the query is used); (ii) Filter with e-value threshold (default 0.05 per chromosome); (iii) Extend the match by adding flanking nucleotides (default 50) up- and down-stream from the match (Ensembl genome database; [http://www.ensembl.org]). Examine all possible extensions of the match within threshold length (default min 70 nt, max 180 nt); (iv) Filter with RNA secondary folding energy threshold (default -25 kcal/mole; RNAfold with options "-p -d2 -noLP" [http://www.tbi.univie.ac.at/RNA]); (v) Filter with minimal base-pairing threshold (default 55% pairing; with 20 gap penalty and 0.5 extension penalty); (vi) Filter with requirement for hairpin-shape secondary structure; (vii) Filter with alignment of precursor sequences (default 56% identity); (viii) Filter with alignment of mature miRNA sequences (default 80% identity); (ix) Filter with maximum number of mismatches in mature miRNA sequences (default 3 nt); (x) Filter with conservation of seed (2-8 nt, required 100% conservation [10]); (xi) Filter with position of mature miRNA on the hairpin (max 4 nt overlap of mature sequence and hairpin loop). In this study we looked at homolog genes which are genes related to each other by descent from a common ancestral DNA sequence. We do not segregate between orthologs, genes in different species that evolved from a common ancestral gene by speciation, and paralogs, genes separated by the event of genetic duplication. miRviewer presents novel miRNAs that are evolutionary conserved based on previously defined features [11]. The parameters used to find these miRNAs are considered stringent as they identify homolog matches after a miRNA has been experimentally confirmed and the default search filters such that it selects 95% of known miRNAs in training genomes (criteria was also based on [11]).

**Figure 3:**
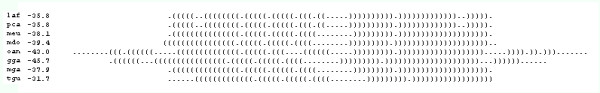
**A partial screenshot of known and novel miR-21 secondary structures**. This view, presented alongside (to the right) of the sequence alignment (presented in Figure 2) allows viewing the predicted secondary structure (based on RNAfold, http://www.tbi.univie.ac.at/RNA) of a specific miRNA among the various organisms. The three letter abbreviations represent the animal and the negative numbers indicate the folding energy (delta G).

On the main web-server page the conservation degree was calculated as the proportion of identical bases. In the alignment interface the mature miRNA sequences are designated by blue highlighting. Outlier nucleotides are colored red. However, we note that in some cases the BLAST searches identify a mature miRNA without the first nucleotide even when the first nucleotide is identical. Thus, some miRNAs might be 'missing' a (colored) first nucleotide. The reason for this perhaps occurs due to the inherent features of the search and should be cautiously taken into consideration. Given that many miRNAs are currently reported to have several isoforms [12-14] we suggest using our web-server in conjugation with the 'deep sequencing view' of miRbase which allows the evaluation of the small changes in mature miRNA nucleotide ends. The secondary structure of each miRNA, presented to the right of each sequence (Figure 3), allows the appreciation of the folding of each miRNA sequence. Close observation of the secondary structure of each miRNA between the various species carries relevant information such as the development of tight or relaxed hairpin structures.

## Methods

### Preparing RNA samples and testing miRNA expression

Brain marmoset RNA was received from Dr. Yona Goldshmit. Rabbit heart sample were received from Dr. Michael Har-Lev. Dolphin epithelium sample was received from Dr. Nadav Shashar. HeLa cell line was used as a positive control for known miRNAs (data not shown). Tissue samples were homogenized prior to RNA extraction. Total RNA was extracted from all samples using TRIzol reagent (Invitrogen) according to the manufacturer's instructions. One microgram of total RNA was used to generate cDNA using the High Capacity Reverse Transcription Kit with random primers (Applied Biosystems) according to manufacturer's instruction and in a final volume of 20 μL. PCR amplification was done using C1000 Thermal Cycler (Bio-Rad) in the following conditions: enzymatic activation at 94°C for 3 min, 30-35 cycles of denaturation at 94°C for 30 s, annealing at 55°C for 30 s and elongation at 72°C for 30 s and then extra 5 min of elongation at 72°C. miRNA-16-1 and miRNA-101-1 species specific primers were obtained from the miRViewer latest version (pre-miR-16-1F: 5'-TCA GCA GTG CCT TAG CAG C-3', pre-miR-16-1R: 5'-CAA CCT TAC TTC AGC AGC AC-3', pre-miR-101-1F: 5'-TGG CTC AGT TAT CAC AGT GC-3', pre-miR-101-1R: 5'-TGC CAT CCT TCA GTT ATC ACA-3'). Total RNA only, of each sample, was used as a negative control for the PCR reaction. Samples were stained with Ethidium Bromide and run in a 2% Agarose gel. Samples were then extracted from the gel using Wizard SV Gel (Promega) according to the manufacturer's instructions and sequenced primed by the PCR primers.

### Sequencing and analysis

Human SupT1 and Hela cell lines were used in the deep sequencing portion. Total RNA was extracted using TRIzol reagent (Invitrogen) and each sample was prepared for deep sequencing following Illumina's Small RNA sample preparation protocol v1.5. Briefly, samples were ligated with 3' and 5' adapters, reverse-transcribed and then PCR amplified. cDNA library was prepared from 93-100 bp PCR products and sequenced in separate lanes on an Illumina Genome Analyzer IIx instrument at the Tel Aviv University Genome High-Throughput Sequencing Laboratory. Adapters were removed from the output files using FASTX-Toolkit (http://hannonlab.cshl.edu/fastx_toolkit), discarding all reads shorter than 16 nucleotides after clipping. Alignment was performed using Burrows-Wheeler Aligner (BWA) ([15]), against a reference of all the predicted miRviewer mature miRNAs sequences with additional three nucleotides flank. Hits were counted when a read aligned perfectly to a predicted miRNA (alignment against the transcript sense strand without any mismatches). Target conservation was calculated by applying TargetScan [10] on the seed region of each predicted miRNA.

## Findings and discussion

miRviewer is a web-server which allows the global overview of miRNAs in the animal kingdom. Our web-server is different and has unique features over other miRNA gene catalogues online. miRortho [16] for example, which contains valuable information on animal miRNAs, does not allow a one screen view of all miRNAs across the animal phyla. Another useful web-server, MapMi [17], does not exhibit an alignment of the miRNA matched sequences. Importantly, miRviewer is based on additional novel miRNA datasets which were identified using our miRNAminer algorithm [7]. Of particular interest, for 27 organisms we report, for the first time, their entire set of annotated homologous miRNAs. Thus, our web-server has both known and predicted miRNAs, clearly differentiated on miRviewer webpage.

**Figure 4:**
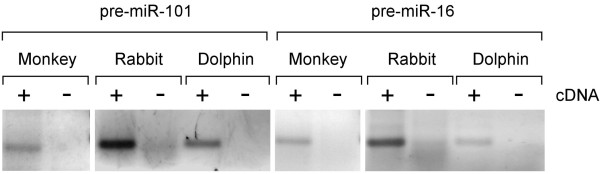
**Confirmation of the expression of two novel miRNAs, miR-101 and miR-16, in monkey (marmoset), rabbit and dolphin**. PCR was used to amplify expressed miRNAs based on presence in miRviewer. Amplified fragments were stained and separated in Agarose gel then excised and sequenced for confirmation (Additional file 1: Figure S1). These miRNAs were identified as part of a very large set of homologous miRNAs using the miRNAminer tool [7] all of which are presented in miRviewer.

In order to confirm a small representation of predicated miRNAs we chose to amplify precursor miR-16 and miR-101 from monkey, rabbit and dolphin total RNA (see Methods and Figure 4). All three mammals showed expression of these miRNAs in the tested tissues. Similarly, when parsing deep sequencing data of two human cell lines for novel miRviewer human mature miRNAs 12 of them were identified to be expressed at more than 10 reads per miRNA (Table 1). Some of these miRNAs have many conserved mRNA target sites suggesting a possible functional role during cellular processes. These two sets of experiments indicate that miRNAminer, and hence miRviewer, represent genuine novel miRNAs and strengthen our comprehensive view of the miRNA world. We caution that by no means have these experiments intended to represent a complete analysis of all predicted miRviewer miRNAs but rather to indicate that future confirmation is feasible. We also note that these results validate not only our predictive algorithm but also the utility of our web-server for species specific primer design.

**Table 1:** Novel miRviewer identified human mature miRNAs discovered using deep sequencing

miRNA Name	Hela	SupT1	Total	Mature Sequence	Seed	Conserved Targets	Conserved Sites
hsa-miR-365-1*	407	901	1308	ACCGAUUUCAAAUGGUGCUA	CCGAUUU	9	9

hsa-miR-652*	180	135	315	GCCCUUUUAACAUUGCACUG	CCCUUUU	486	521

hsa-miR-664b	131	98	229	AACACCAUUGUCACACUCCA	ACACCAU	195	204

hsa-miR-301a*	79	27	106	CAACCCUAGGAGAGGGUGCCAUUC	AACCCUA	70	72

hsa-miR-513a	0	65	65	AGGGGUUCACCGAGCAACAUUCG	GGGGUUC	88	110

hsa-miR-128-1*	21	37	58	ACGGGUAUUCUUGGGUGGAUAA	CGGGUAU	2	2

hsa-miR-152*	23	0	23	GGAAACAUUUCUGCACAAACUAG	GAAACAU	360	375

hsa-miR-1249*	1	20	21	GCUGGUAAAAUGGAACCAAA	CUGGUAA	146	151

hsa-miR-103-1*	13	4	17	GGCUUCUUUACAGUGCUGCCUUG	GCUUCUU	392	412

hsa-miR-2145	12	4	16	GCGACGAGCCCCUCGCACAAACC	CGACGAG	1	1

hsa-miR-107*	1	14	15	AGCUUCUUUACAGUGUUGCCUUG	GCUUCUU	392	412

hsa-miR-203*	11	0	11	GUGGAUAUUCCUUCUAUGUUUA	UGGAUAU	116	119

* refers to the minor miRNA isoform (based on miRbase)

Future developments of miRviewer include incorporation of additional animal genomes as they are sequenced and annotated. Adding further layers of information such as performing statistical analysis of each miRNA family and allowing the user to choose an animal to display as a reference set (the current reference set is based on human). We would like to note that the main webpage would remain the same for simplicity and ease of use. The additional layers of information would be added as hyperlink.

## Conclusions

The miRviewer web-server, presented here, encompasses all known and predicted miRNAs of fully annotated animal genomes. miRviewer's interface provides the user with a graphic outlook of the current miRNA world together with additional information such as sequence alignments and secondary structure. miRviewer is available at: http://people.csail.mit.edu/akiezun/miRviewer

## Availability and requirements

miRviewer is available online at: http://people.csail.mit.edu/akiezun/miRviewer

## Competing interests

The authors declare that they have no competing interests.

## Authors' contributions

NS, AK and SA conceived and designed the web-server. AK and SA wrote and ran the algorithms to search for homologous miRNAs and constructed the web-server. SM and NV performed the experimental validation. OI performed the deep sequencing data analysis. All authors read, contributed to and approved the final manuscript.

## Supplementary Material

Additional file 1 **Figure S1**. Sequencing results of pre-miRNA-16-1 and pre-miRNA-101-1 of **(A) **Rabbit and **(B) **Marmoset PCR samples, compared to miRViewer (and miRNAminer) predictions. Click here for file
