# Supplementary figures and images for: miRviewer: a multispecies microRNA homologous viewer
Source: BMC Res Notes. 2012 Feb 13;5:92. doi: 10.1186/1756-0500-5-92 (PMC3292992; doi:10.1186/1756-0500-5-92)

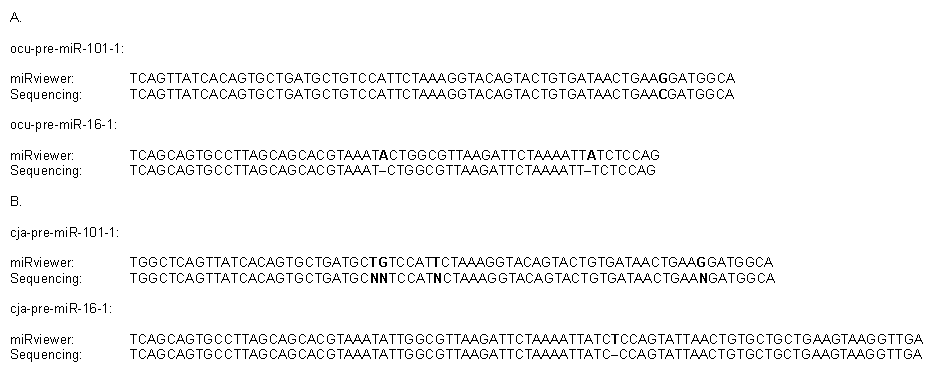

Supplement: Additional file 1 — Figure S1. Sequencing results of pre-miRNA-16-1 and pre-miRNA-101-1 of (A) Rabbit and (B) Marmoset PCR samples, compared to miRViewer (and miRNAminer) predictions. [file 1756-0500-5-92-S1.DOC]
